# Genome Analysis of *Pseudomonas fluorescens* PCL1751: A Rhizobacterium that Controls Root Diseases and Alleviates Salt Stress for Its Plant Host

**DOI:** 10.1371/journal.pone.0140231

**Published:** 2015-10-09

**Authors:** Shu-Ting Cho, Hsing-Hua Chang, Dilfuza Egamberdieva, Faina Kamilova, Ben Lugtenberg, Chih-Horng Kuo

**Affiliations:** 1 Institute of Plant and Microbial Biology, Academia Sinica, Taipei, Taiwan; 2 Institute for Landscape Biogeochemistry, Leibniz Centre for Agricultural Landscape Research (ZALF), Eberswalder str. 84, Müncheberg, Germany; 3 Koppert Biological Systems, Veilingweg 14, 2651 BE Berkel en Rodenrijs, the Netherlands; 4 Institute of Biology, Sylvius Laboratory, Leiden University, Leiden, the Netherlands; 5 Molecular and Biological Agricultural Sciences Program, Taiwan International Graduate Program, National Chung Hsing University and Academia Sinica, Taipei, Taiwan; 6 Graduate Institute of Biotechnology, National Chung Hsing University, Taichung, Taiwan; University of Wisconsin-Milwaukee, UNITED STATES

## Abstract

*Pseudomonas fluorescens* PCL1751 is a rod-shaped Gram-negative bacterium isolated from the rhizosphere of a greenhouse-grown tomato plant in Uzbekistan. It controls several plant root diseases caused by *Fusarium* fungi through the mechanism of competition for nutrients and niches (CNN). This mechanism does not rely on the production of antibiotics, so it avoids the concerns of resistance development and is environmentally safe. Additionally, this bacterium promotes plant growth by alleviating salt stress for its plant host. To investigate the genetic mechanisms that may explain these observations, we determined the complete genome sequence of this bacterium, examined its gene content, and performed comparative genomics analysis with other *Pseudomonas* strains. The genome of *P*. *fluorescens* PCL1751 consisted of one circular chromosome that is 6,143,950 base-pairs (bp) in size; no plasmid was found. The annotation included 19 rRNA, 70 tRNA, and 5,534 protein-coding genes. The gene content analysis identified a large number of genes involved in chemotaxis and motility, colonization of the rhizosphere, siderophore biosynthesis, and osmoprotectant production. In contrast, the pathways involved in the biosynthesis of phytohormones or antibiotics were not found. Comparison with other *Pseudomonas* genomes revealed extensive variations in their genome size and gene content. The presence and absence of secretion system genes were highly variable. As expected, the synteny conservation among strains decreased as a function of phylogenetic divergence. The integration of prophages appeared to be an important driver for genome rearrangements. The whole-genome gene content analysis of this plant growth-promoting rhizobacterium (PGPR) provided some genetic explanations to its phenotypic characteristics. The extensive and versatile substrate utilization pathways, together with the presence of many genes involved in competitive root colonization, provided further support for the finding that this strain achieves biological control of pathogens through effective competition for nutrients and niches.

## Introduction

The bacterial genus *Pseudomonas* contains diverse species that inhabit soil, water, and plant surfaces [1,2]. In addition, many *Pseudomonas* species isolated from saline soils were found to exhibit beneficial effects on plant development [3–7]. These plant growth-promoting rhizobacteria (PGPR) use direct or indirect mechanisms to stimulate plant growth, to increase plant stress tolerance, and to protect plants from pathogens [2,8,9]. These mechanisms include: (i) promoting the nutrient availability, such as increasing phosphate solubility [10–12], (ii) production of phytohormones, such as auxin, to stimulate growth [13,14], (iii) reduction of stress-induced ethylene production [15,16], (iv) production of osmoprotectants [17], (v) production of antifungal metabolites [1,18,19], (vi) induction of plant systemic resistance [20,21], and (vii) competition for nutrients and niches (CNN) [22,23].

The strain *P*. *fluorescens* PCL1751 was isolated from a screen for rhizobacteria with enhanced competitive plant root tip colonization [22]. Phenotypic characterization of this strain revealed that it could provide protection against the disease foot and root rot in tomato caused by the fungal pathogen *Fusarium oxysporum* [22] and in cucumber caused by *F*. *solani* [23]. These biocontrol traits appeared to be the result of its strong competition ability for nutrients and niches [22,23]. Because this CNN mechanism is based on multiple traits and does not involve antibiotic production, it has the additional advantage that it does not raise the concern of resistance development. Therefore, *P*. *fluorescens* PCL1751 was considered to be safer and more practical than biocontrol strains that exhibit direct antagonistic activities against phytopathogens. Moreover, *P*. *fluorescens* PCL1751 has the advantage that it is salt tolerant and significantly promotes plant growth in salinated soil [23].

To better understand the biology of *P*. *fluorescens* PCL1751, the main goals of this study were to determine the complete genome sequence of this bacterium, investigate its gene content, and infer the link between its gene content and biological traits. Additionally, comparative genomics analysis within the *P*. *fluorescens* group may provide further insights into the genome evolution among these diverse soil bacteria that often exhibit plant-growth promoting abilities [24–31].

## Methods

### Genome Sequencing, Assembly, and Annotation

The strain *P*. *fluorescens* PCL1751 was obtained from the collection of the Institute of Biology, Leiden University, the Netherlands (contact: Prof. Ben Lugtenberg). The procedures for genome sequencing, assembly, and annotation are based on those described in our previous studies [32–36]. Briefly, the bacterium was cultured in standard Tryptic Soy Broth (Becton, Dickinson and Company) medium prior to DNA extraction by using the Wizard Genomic DNA purification Kit (Promega) according to manufacturer’s protocol. Two libraries were constructed and sequenced by a commercial service provider (Yourgene Bioscience, New Taipei City, Taiwan) using the Illumina MiSeq platform (Illumina), including one paired-end library (~481 bp insert; 351 + 251 bp reads; ~5.0 Gb raw reads; deposited at the NCBI SRA database under the accession number SRR2285883) and one mate-pair library (~4.2 kb insert; 251 bp * 2 reads; ~1.3 Gb raw reads; accession number SRR2285884).

The *de novo* assembly was performed using ALLPATHS-LG release 42781 [37] according to the user’s manual. The initial assembly was iteratively improved by mapping the raw reads to the contigs using Burrows-Wheeler Aligner (BWA) version 0.6.2 [38], programmatically checked using the MPILEUP program in SAMTOOLS package version 0.1.18 [39], and visually inspected using Integrative Genomics Viewer (IGV) version 2.1.24 [40]. The scaffolding across repetitive regions was confirmed by PCR and the gaps were filled using reads extending over the contig ends. The sequence polymorphisms among rRNA operons were resolved using the mate-pair reads anchored at adjacent unique regions. The iterative process was repeated until the entire circular chromosome sequence was determined.

The programs RNAmmer [41], tRNAscan-SE [42], and PRODIGAL [43] were used for gene prediction with the settings specified for bacterial genomes in the user’s manual. For each protein-coding gene, the gene name and product description were initially annotated based on the homologous genes in *P*. *fluorescens* SBW25 [25] as identified by OrthoMCL [44] with a BLASTP [45] e-value cutoff of 1e-15 and an inflation value of 1.5. Subsequently, BLASTP searches against the NCBI non-redundant (nr) protein database [46] and the Kyoto Encyclopedia of Genes and Genomes (KEGG) database [47,48] were used to assist manual curation of the annotation. The final annotated chromosome was plotted using CIRCOS [49] to show gene locations, GC-skew, and GC content. The pathway analysis was based on the categories defined in the KEGG Pathway database and manual inspection of annotation evidences from NCBI BLASTP search results, as well as the homologous genes in other *Pseudomonas* genomes and their description in the literature (Table 1).

**Table 1:** Genome characteristics.

ID	Strain	GenBank accession	Size (bp)	G+C (%)	rRNA genes (operons)	tRNA genes	Protein-coding genes	Plasmids	Property	Reference
PCL1751	*P*. *fluorescens* PCL1751	CP010896	6,143,950	60.4	19 (6)	70	5,534	-	Biocontrol by competition for nutrients and niches, plant growth-promotion, and increase of plant salt stress tolerance	This study
SBW25	*P*. *fluorescens* SBW25	AM181176	6,722,539	60.5	16 (5)	68	5,921	-	Plant growth-promotion	[25]
A506	*P*. *fluorescens* A506	CP003041	5,962,570	60.0	19 (6)	69	5,267	1	Biocontrol	[26]
UK4	*P*. *fluorescens* UK4	CP008896	6,064,456	60.1	19 (6)	68	5,178	-	Biofilm-forming and amyloid-producing	[29]
Pf0-1	*P*. *fluorescens* Pf0-1	CP000094	6,438,405	60.5	19 (6)	73	5,722	-	Soil-dwelling commensal	[25]
UW4	*P*. sp. UW4	CP003880	6,183,388	60.1	22 (7)	72	5,423	-	Plant growth-promotion	[28]
F113	*P*. *fluorescens* F113	CP003150	6,845,832	60.8	16 (5)	66	5,862	-	Biocontrol by secondary metabolites production	[27]
Pf-5	*P*. *protegens* Pf-5	CP000076	7,074,893	63.3	16 (5)	71	6,108	-	Biocontrol by antibiotics production	[24]
CHA0	*P*. *protegens* CHA0	CP003190	6,867,980	63.4	15 (5)	68	6,115	-	Biocontrol by antibiotics production	[30]
PA23	*P*. *chlororaphis* PA23	CP008696	7,122,173	62.6	16 (5)	68	6,179	-	Biocontrol by antibiotics production	[31]
DC3000	*P*. *syringae* DC3000	AE016853	6,397,126	58.4	15 (5)	63	5,482	2	Phytopathogenic	[50]

### Comparative Genomics Analyses

For comparative genomics analyses, we focused on the strains within the *P*. *fluorescens* group of which the complete genome sequence is available (Table 1). Additionally, *P*. *syringae* pv. *tomato* str. DC3000 [50] was included as the outgroup. For comparison of their gene content, we utilized the original annotation included in the GenBank files downloaded from the NCBI nucleotide database. The homologous gene clusters were identified by using OrthoMCL with the same parameters as described above. Genes involved in secretion systems were manually curated based on the KEGG nomenclatures as listed in the ‘Bacterial Secretion System’ page of the KEGG Pathway Maps. For analysis of prophages in these genomes, we utilized the PHAST web server [51] to identify the putative prophages and curated the results manually.

For molecular phylogenetic inference, we aligned the protein sequences of the 2,374 homologous gene clusters that contain exactly one orthologous gene from each of the 11 genomes compared using MUSCLE version 3.8.31 [52] with the default settings. The final concatenated alignment contains a total of 783,597 amino acid sites. A maximum likelihood phylogeny was inferred using PHYML version 20120412 [53]. The proportion of invariable sites and the gamma distribution parameter were estimated from the data set and the number of substitute rate categories was set to four. Bootstrap supports were estimated based on 1,000 replicates generated by the SEQBOOT program of PHYLIP v3.69 [54], followed by PHYML inference as described above for each replicate and the extended majority rule (MRe) consensus phylogeny inference by the CONSENSE program of PHYLIP.

For pairwise genome alignments, we utilized MUMmer version 3.23 [55]. To reduce spurious hits, we increased the minimum match length (option ‘-l’) to 40 from the default setting of 20. The genome-wide sequence similarities were calculated based on the 2,374 conserved single-copy genes using the DNADIST and PROTDIST programs of PHYLIP.

### Substrate Utilization Test

Growth of *P*. *fluorescens* PCL1751 on various carbon sources was tested on solid BM medium [22] containing 0.2–0.4% of 16 different carbon sources. A Petri dish without the added carbon source was included as a control. A small amount of *P*. *fluorescens* PCL1751 cells were streaked to purity on the solid media and incubated at 28°C. The appearance of clearly visible single colonies was taken as the criterion for growth.

## Results and Discussion

### Genome Characteristics

**Fig 1:**
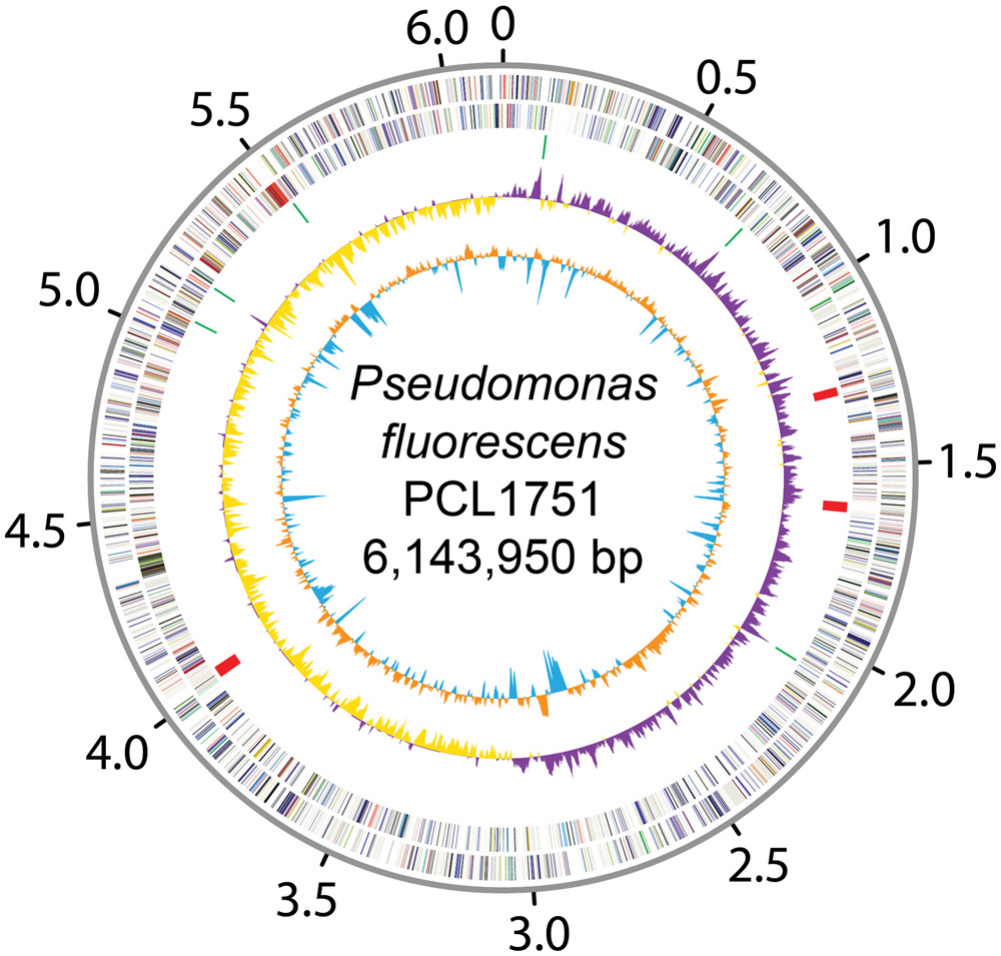
Genome map of *Pseudomonas fluorescens* PCL1751. Rings from the outside in: (1) scale marks (unit: Mb), (2 and 3), protein-coding genes on the forward and reverse strand, respectively (color-coded by the functional categories), (4) rRNA gene clusters (green) and prophages (red), (5) GC skew (positive: purple; negative: yellow), and (6) GC content (above average: orange; below average: blue).

The complete genome of *P*. *fluorescens* PCL1751 contains a circular chromosome that is 6,143,950 bp in size with a G+C content of 60.4% (Fig 1 and Table 1). No plasmid was found. This sequence has been deposited at DDBJ/EMBL/GenBank under the accession number CP010896. The first version of annotation includes 19 rRNA genes (organized into six operons, the first of which contains an extra 5S rRNA gene), 70 tRNA genes, and 5,534 protein-coding genes. The protein-coding genes have an average length of 995.6 bp and account for 89.5% of the chromosome.

### Chemotaxis, Motility, Adhesion, and Other Aspects of Root Colonization

As expected for a rhizobacterium that exhibits strong competitive colonization ability of plant roots [22], we identified the genes required for chemotaxis, motility, and adhesion. Details on the roles of motility and chemotaxis in root colonization have been reviewed previously [2,56,57]. For chemotaxis, both the Che (*cheA*, *cheB*, *cheR*, *cheW*, *cheY*, and *cheZ*) and the Wsp (*wspA*, *wspB*, *wspC*, *wspD*, *wspE*, *wspF*, *wspR*) systems for signal transduction are present. Notably, 33 copies of the methyl-accepting chemotaxis protein genes (*mcp*) were found, suggesting that this bacterium has a wide range of trans-membrane sensor proteins for different signals. For motility, the genes involved in the regulation (*fleQ*, *fleR*, and *fleS*), biosynthesis (*flhA*, *flhB*, *flhF*, *flhG*, *fliP*, *fliQ*, and *fliR*), structure (*flgA*, *flgB*, *flgC*, *flgD*, *flgE*, *flgF*, *flgG*, *flgH*, *flgI*, *flgJ*, *flgK*, *flgL*, *fliC*, *fliD*, *fliE*, *fliF*, *fliG*, *fliH*, *fliI*, *fliJ*, *fliK*, *fliL*, *fliM*, *fliN*, *fliO*, *fliS*, and *fliT*), and motor (*motA* and *motB*) components of flagella were found.

As an adhesive structure, pili play a crucial role in host-bacteria interactions [58,59]. The genes for type IV pilus system (*pilA*, *pilC*, *pilD*, *pilE*, *pilF*, *pilG*, *pilH*, *pilI*, *pilJ*, *pilM*, *pilN*, *pilO*, *pilQ*, *pilT*, *pilV*, *pilX*) were found in our gene content survey. Furthermore, several genes involved in biofilm formation were found, including those responsible for polysaccharide transport (*pelA*, *pelB*, *pelC*, *pelD*, *pelF*, *pelF*, and *pelG*), adhesin production (*pgaA*, *pgaB*, *pgaC*, and *pgaD*), and 19 GGDEF domain proteins. The GGDEF domain is associated with diguanylate cyclase activity, which is involved in cyclic di-GMP signaling for biofilm formation and persistence [60]. Finally, the malate dehydrogenase gene (*mqo*) was also found in the genome of *P*. *fluorescens* PCL1751. This gene is involved in growth on organic acids (including malic acid, succinic acid, and citric acid) and is required for the colonization of the tomato root by *P*. *fluorescens* WCS365 [56].

### Utilization of Organic Carbon Sources

**Fig 2:**
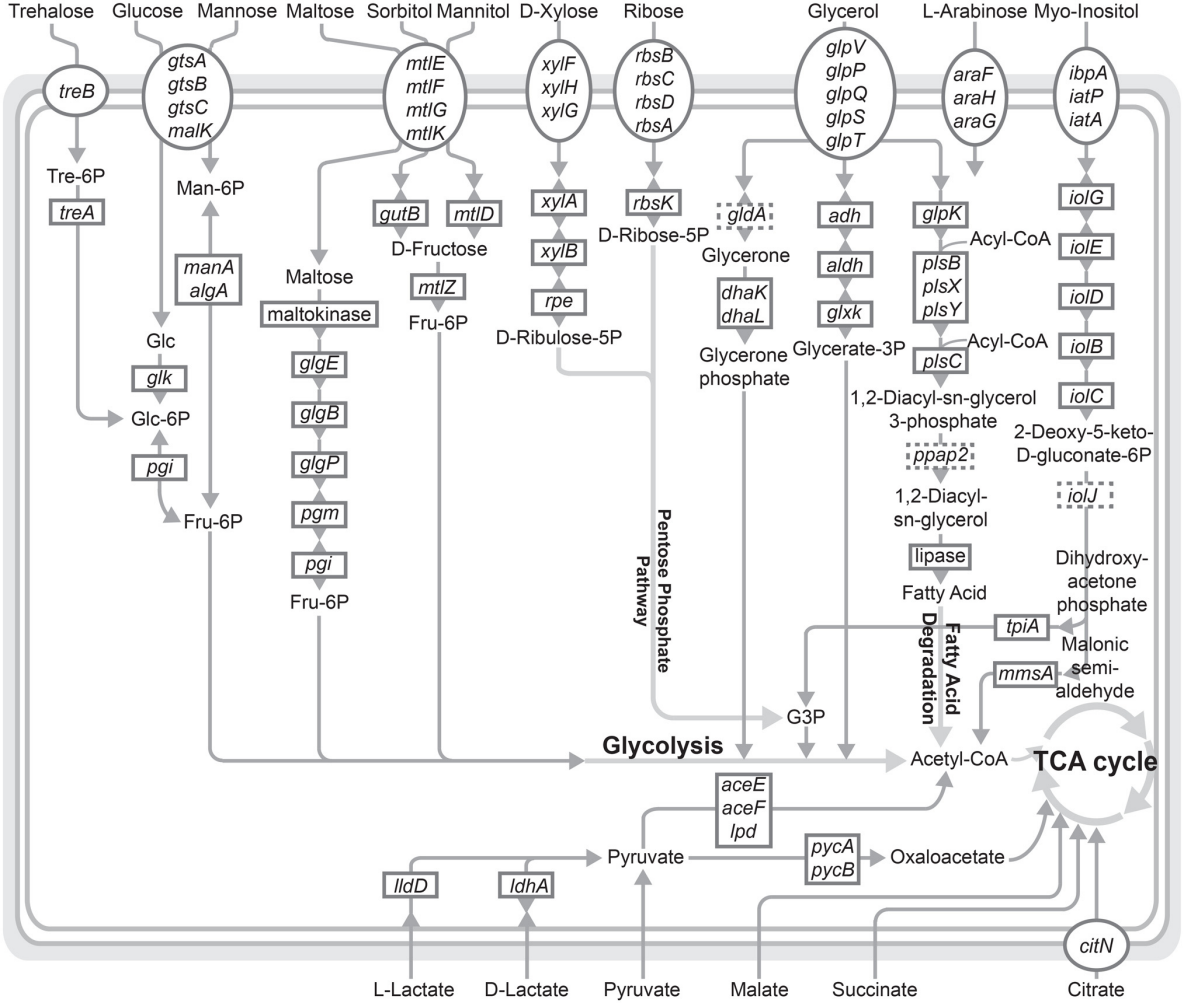
Selected metabolic pathways of *Pseudomonas fluorescens* PCL1751. This figure provides a visual summary of the gene content analysis described in the main text. Boxes drawn with dotted lines indicate the genes missing in the annotation.

A visual summary of selected metabolic pathways inferred from the gene content analysis of *P*. *fluorescens* PCL1751 is presented in Fig 2. This bacterium possesses a diverse array of genes involved in the utilization of organic carbon sources available in the rhizosphere, which is consistent with its superior growth on tomato root exudate compared to other strains [22]. Based on the previous characterization of tomato root exudates, citric acid, malic acid, and lactic acid are the most abundant organic acids [61]. The metabolic pathway inference suggested that these organic acids could be readily incorporated into the tricarboxylic acid (TCA) cycle. Furthermore, *P*. *fluorescens* PCL1751 possesses genes for the uptake and conversion of other carbon sources, such as various sugars (trehalose, glucose, mannose, maltose, xylose, ribose, and arabinose), sugar alcohols (sorbitol and mannitol), *myo*-inositol, and glycerol, to be utilized through the pentose phosphate pathway and glycolysis (Fig 2).

These pathway predictions were validated through growth experiments. Positive growth of *P*. *fluorescens* PCL1751 was recorded after 24 hours on casamino acids, lactic acid, and succinic acid. After 48 hours, positive growth was observed on all other tested carbon sources, namely arabinose, citric acid, glucose, glycerol, malic acid, maltose, mannitol, mannose, *myo*-inositol, ribose, sorbitol, trehalose, and xylose. No growth was observed on the control plate.

### Plant Growth Promotion and Protection

For phytohormone-related genes, one 1-aminocyclopropane-1-carboxylate (ACC) deaminase gene (*acdS*) was found, indicating that this bacterium could act as a sink for ACC and lower the level of the plant stress hormone ethylene. The indole-3-acetic acid (IAA) biosynthesis pathway was not found, suggesting that the plant growth-promoting property of this bacterium is not modulated through auxin production. This finding is consistent with the previous observation that the IAA production of this bacterium was not detectable [22,23].

**Fig 3:**
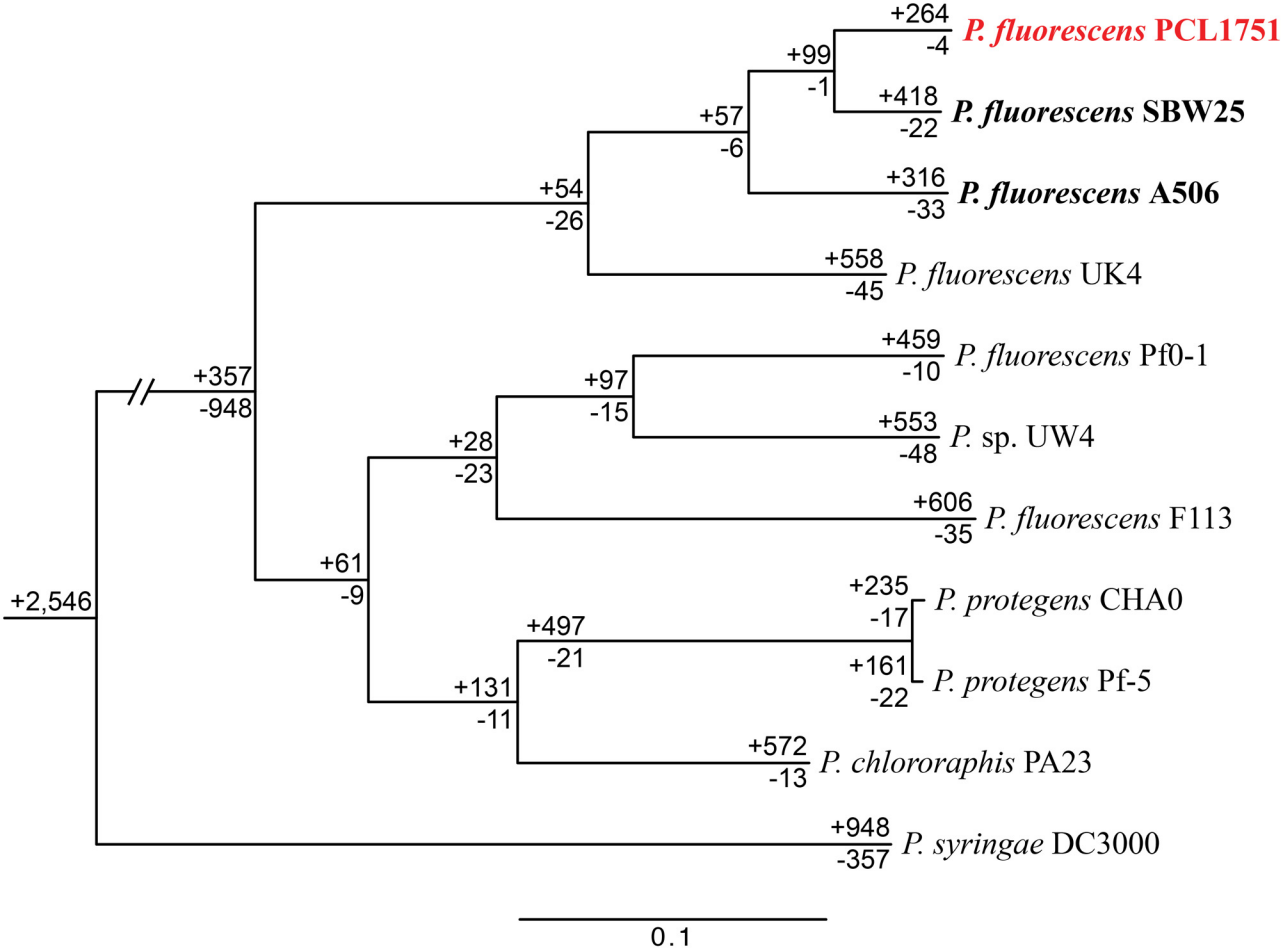
Distribution pattern of homologous gene clusters. The maximum likelihood phylogeny was inferred from the concatenated protein alignment of 2,374 single-copy genes shared by all strains (with 783,597 aligned amino acids). All internal nodes received 100% bootstrap support based on 1,000 re-sampling and maximum likelihood inference. The numbers above each branch and proceeded by a ‘+’ sign indicate the numbers of homologous gene clusters that are uniquely present in all daughter lineages; the numbers below each branch and proceeded by a ‘-‘ sign indicate the numbers of homologous gene clusters that are uniquely absent.

*P*. *fluorescens* PCL1751 is able to utilize chrome azurol S (CAS) in the bioassay on detection of siderophores. Consistent with this observation, several siderophore biosynthesis pathways were found, such as those for pyoverdines (*pvdA*, *pvdE*, *pvdF*, *pvdG*, *pvdH*, *pvdI*, *pvdJ*, *pvdM*, *pvdN*, *pvdO*, *pvdP*, *pvdQ*, and *pvdS*) and pyochelin (*pchA*, *pchB*, *pchC*, *pchD*, *pchH*, *pchI*, *pchK*, *pchP*, and *pchR*). Pyoverdines are a diverse group of fluorescent siderophores produced by pseudomonads and facilitate iron uptake of these bacteria [62,63]. Pyochelin facilitates the acquisition of various metal ions, including iron, copper, and zinc [64]. Furthermore, we found 47 TonB-dependent receptors (TBDRs) and associated genes, indicating that *P*. *fluorescens* PCL1751 may be highly effective in the competitive acquisition of iron and cofactors [65]. Finally, the genes for pyrroloquinoline quinone (PQQ) production (*pqqA*, *pqqB*, *pqqC*, *pqqD*, *pqqE*, *pqqF*, *pqqH*, and *pqqI*) and PQQ-dependent glucose dehydrogenase were found. One previous study on 118 *P*. *fluorescens* strains revealed that the ability for inorganic phosphate solubilization within this species complex is linked to the phylogenetic affiliation [66]. The presence of these genes, together with the close evolutionary relationship between *P*. *fluorescens* PCL1751 and other strains exhibiting strong inorganic phosphate solubilization ability (Fig 3), suggests that PCL1751 is likely to possess such ability as well.

One notable property of *P*. *fluorescens* PCL1751 among PGPR is its high salt tolerance and the ability to promote plant growth in salinated soil [23]. Consistent with these observations, we identified a large number of genes related to osmoprotectant production. For example, alginate is an exopolysaccharide that protects fluorescent *Pseudomonas* against desiccation [67] and we found the associated genes (*algA*, *algB*, *algC*, *algD*, *algE*, *algF*, *algG*, *algI*, *algJ*, *algK*, *algL*, *algQ*, *algR*, *algU*, *algW*, *algX*, *algZ*, and *kinB*). Additionally, genes involved in the biosynthesis and accumulation of other osmoprotectants were found, such as those for carnitine (*opuCA*, *opuCB*, and *opuCC*), choline (*choV*, *choW*, and *choX*), glycine betaine (*gbsA*), proline (*laaA*, *pip*, *proP*, *proV*, *proW*, *proX*, *proY*, *prpA*, *pupA*, and *pupP*), and trehalose (*treA*, *treB*, *treR*, *treS*, *treY*, and *treZ*).

Although *P*. *fluorescens* PCL1751 has been shown to be effective in protecting plants against fungal pathogens, laboratory co-culture of *P*. *fluorescens* PCL1751 with various fungi found no direct antagonistic activity [22,23]. Moreover, no production of chitinase, cellulose, glucanase, or hydrogen cyanide (HCN) was detected for *P*. *fluorescens* PCL1751 [22,23]. These results confirmed that the mechanism for fungal disease suppression by *P*. *fluorescens* PCL1751 does not rely on the anti-fungal compound production pathways found in other closely related *Pseudomonas* strains, such as those related to pyoluteorin in strain Pf-5 [68] or 2,4-diacetylphloroglucinol and HCN in strains F113 [27] and CHA0 [69]. Indeed, we did not find those pathways in the *P*. *fluorescens* PCL1751 genome.

### Comparative Genomics of *P*. *fluorescens* and Related Species

Homologous gene identification among the strains belonging to the *P*. *fluorescens* group found 2,903 gene clusters conserved among these strains (Fig 3; including 2,546 clusters shared by all 11 genomes analyzed and 357 clusters absent in the outgroup *P*. *syringae*). The estimate is close to that from a previous comparative genomics analysis of the *P*. *fluorescens* group [26]. These core genes accounted for only about half of the genes in each genome, suggesting that the genetic diversity in terms of the gene content, rather than sequence divergence, is quite high within this group of bacteria. Furthermore, the genome size also varied considerably with the number of protein-coding genes ranged from 5,178 in UK4 to 6,179 in PA23. These observations suggest that the genomes of these rhizobacteria are highly dynamic and have been shaped by extensive gene gains and losses. The *P*. *putida* group, which shared similar ecological niches with the *P*. *fluorescens* group, was estimated to have a core genome of 3,185 genes, while the estimates for the pathogenic species *P*. *syringae* and *P*. *aeruginosa* were 3,456 and 4,653, respectively [26]. These differences may be partly explained by the complex issues involved in defining species boundaries in bacteria. Additionally, the degree of dependence on eukaryotic hosts may also have contributed to the differences in the genome characteristics of these species [70–72].

**Fig 4:**
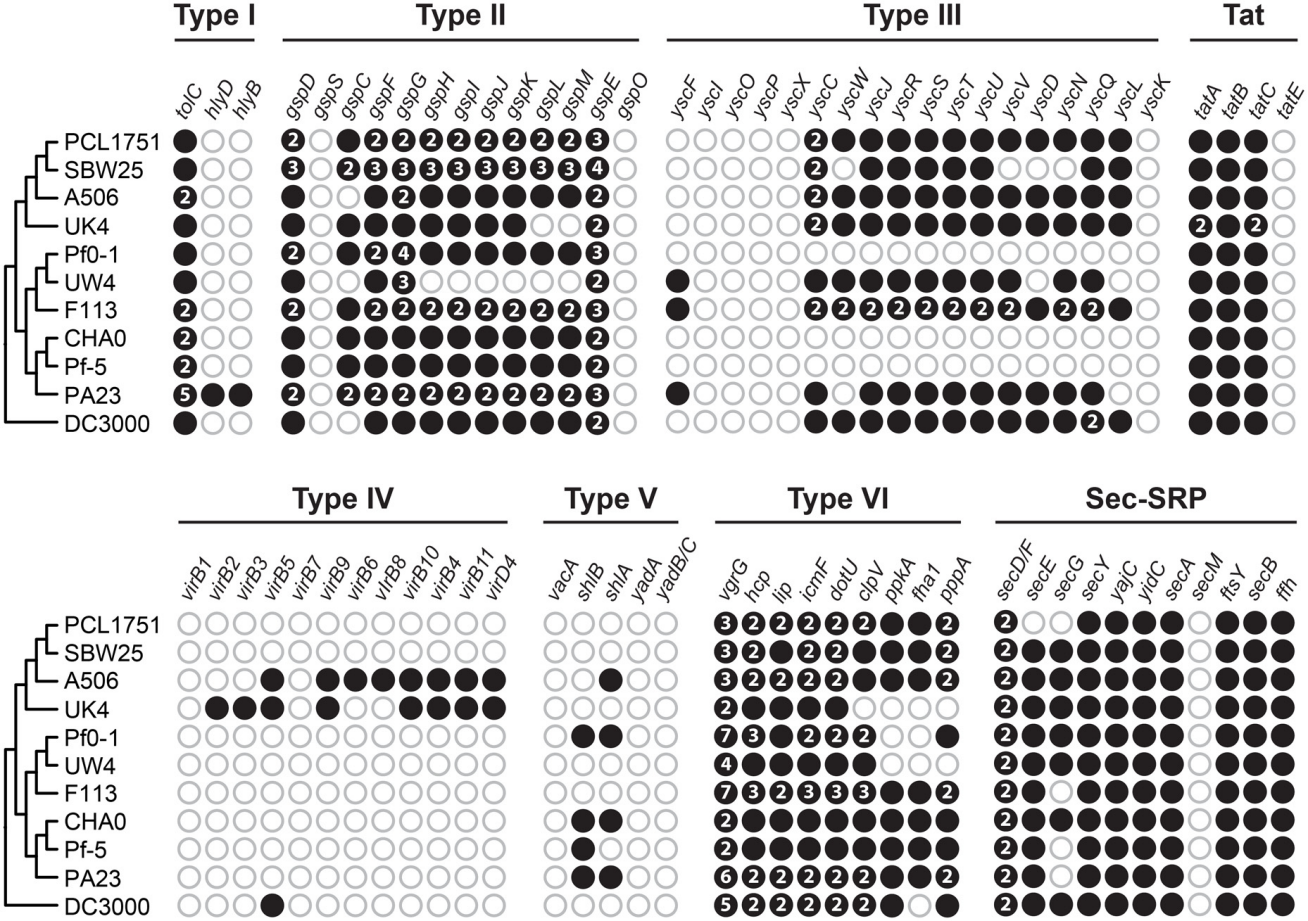
Secretion system gene clusters. The figure provides a visual summary of the presence (filled circles) and absence (empty circles) of genes involved in secretion systems in these genomes. Multi-copy genes are labeled by their copy number inside the filled circles.

Of the genes that exhibited variable patterns of presence and absence in these bacteria, the secretion systems are among the most notable ones due to their functional roles in the interaction with other microbes and the plant hosts [73]. Our results indicated that the type III secretion system (T3SS) genes are entirely absent in strains Pf0-1, CHA0, and Pf-5 (Fig 4; also see ref. [26]). Although the genes *yscI*, *yscO*, *yscP*, *yscX*, and *yscK* were not found in any of the *Pseudomonas* genomes examined, the T3SS in *P*. *syringae* DC3000 has been shown to be functional [50], suggesting that these genes are not essential or have been functionally replaced by other genes. In the previous characterization of SBW25-derived strains, the T3SS mutant Rhi19 (*hrcR*::*Tn5*; KEGG Orthology ID K03226; alternative gene names: *yscR*, *sctR*, or *rscR*) was shown to be normal in tomato root tip colonization when tested alone but exhibited 10-fold reduction when competing against the parental strain. The authors explained this result by hypothesizing that the wild type cells use the T3SS needle to suck nutrients from the plant host [74]. Because the T3SS gene *yscR* are also present in PCL1751, this hypothesis can be extended to this strain.

The type VI secretion system (T6SS) has been shown to be important for competition between *Agrobacterium* and *Pseudomonas* [75]. Most of the *Pseudomonas* genomes examined here possess the complete gene set for the T6SS, suggesting that this secretion system may be important for their ecological niches. Finally, the type I, IV, and V secretion system genes appeared to be incomplete in these genomes.

**Fig 5:**
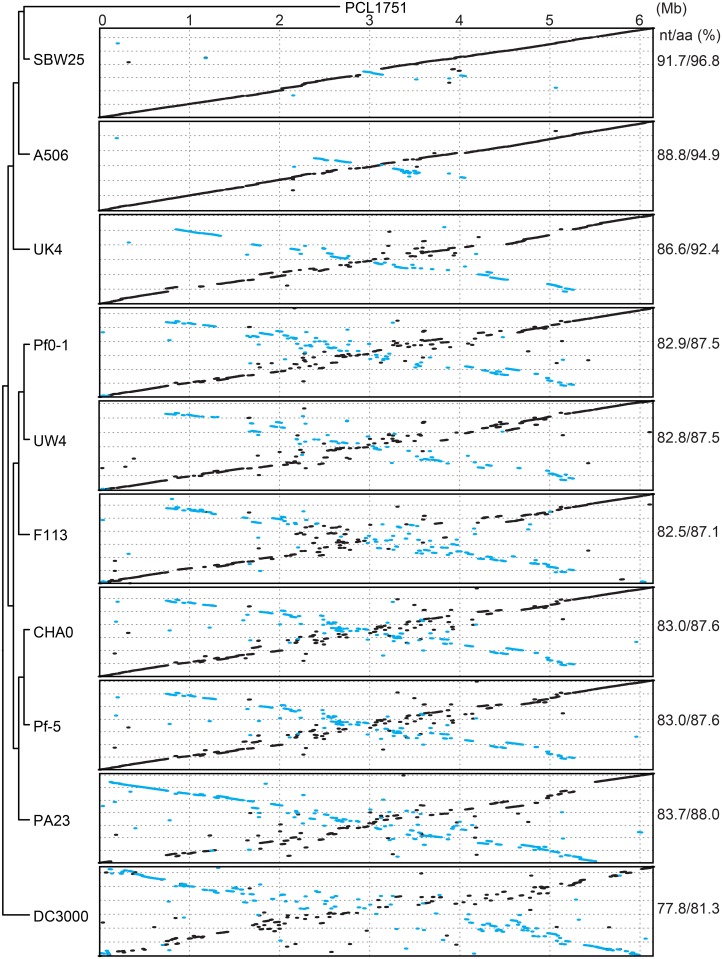
Genome alignments. Pairwise genome alignments between *Pseudomonas fluorescens* PCL1751 and other related strains. The nucleotide (nt) and amino acid (aa) sequence similarities are calculated based on the concatenated alignment of 2,374 single-copy genes shared by all strains.

Based on the molecular phylogeny inferred using the 2,374 single-copy genes shared by all strains, *P*. *fluorescens* PCL1751 is most closely related to SBW25 (Fig 3). This result places PCL1751 in the third sub-clade within the *P*. *fluorescens* group [26]. Our genome alignments (Fig 5) indicated that the chromosomal organization was largely conserved within this subclade (i.e., among strains PCL1751, SBW25, and A506), which share genome-wide sequence similarities of ~89% and ~95% at the nucleotide (nt) and amino acid (aa) level, respectively. Strain UK4 appeared to fall outside of this sub-clade and exhibited a lower level of synteny conservation.

**Fig 6:**
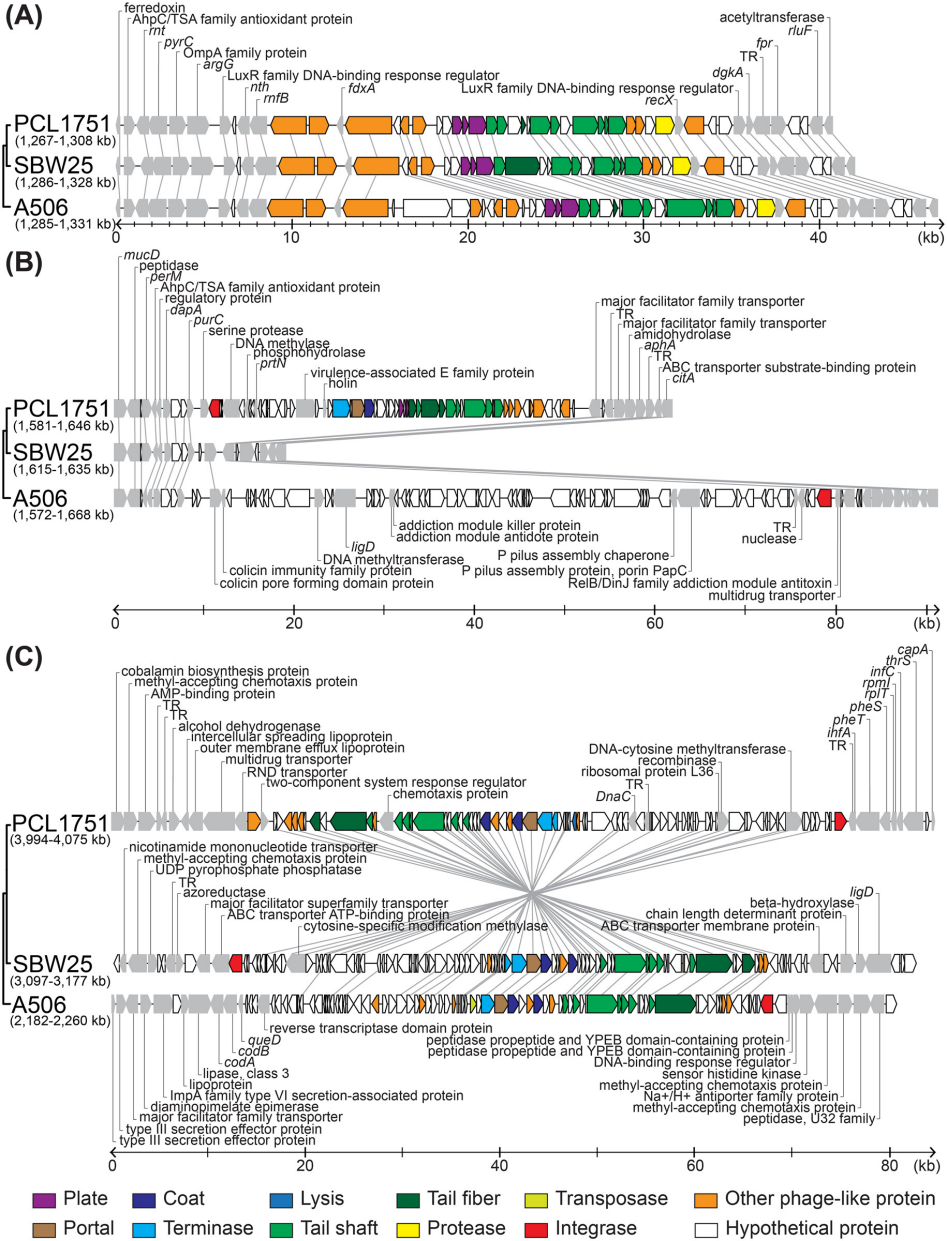
Synteny of prophage insertion sites. Gene organization near the three prophage insertion sites in the *Pseudomonas* ***fluorescens*** PCL1751 genome and the syntenic regions in the two closely related strains. Putative homologs are linked by thin gray lines.

For more detailed comparisons of the chromosomal organization among PCL1751, SBW25, and A506, we found several types of prophage insertions. In the first example (Fig 6A), one prophage appeared to have integrated into the common ancestor of these three strains and remained stable, thus the syntenic region involving the phage sequence and neighboring genes are highly conserved among the three genomes. In the second example (Fig 6B), one ~50 kb prophage insertion was specific to PCL1751 and absent in SBW25. Intriguingly, in the corresponding region of the A506 genome, we found one ~70 kb segment of DNA that lacks identifiable homology to the other genomes, suggesting that this site may be an insertional hotspot. In the third example (Fig 6C), one prophage was found in all three genomes yet was located in different regions in each, suggesting that this prophage may had been active in transposition after the initial integration or had been acquired independently. Taken together, these findings indicate that prophage insertions have contributed to the evolution of chromosomal organization in these bacteria.

## Conclusions

By determining the complete genome sequence of *P*. *fluorescens* PCL1751, this study allowed for the investigation of its entire gene content. The genes that are present in the genome correspond well with previous phenotypic characterizations with respect to biocontrol of plant pathogens and plant growth promotion. Furthermore, the absence of certain genes (e.g., production of auxin or antibiotics) could be confirmed and are equally informative. The comparative genomics analyses with other related strains revealed extensive genetic variations among these diverse plant-associated rhizobacteria with the potential for biotechnology applications. Finally, this study provides a foundation for future genetic investigation of this bacterium and its molecular interactions with the plant hosts.
